# Research Progress on the Application of Triboelectric Nanogenerators for Wind Energy Collection

**DOI:** 10.3390/mi14081592

**Published:** 2023-08-13

**Authors:** Jin Yan, Zhi Tang, Naerduo Mei, Dapeng Zhang, Yinghao Zhong, Yuxuan Sheng

**Affiliations:** 1 College of Shipping and Maritime Transportation, Guangdong Ocean University, Zhanjiang 524088, China; 2 Shenzhen Research Institute, Guangdong Ocean University, Shenzhen 518120, China; 3 College of Mechanical Engineering, Guangdong Ocean University, Zhanjiang 524088, China

**Keywords:** triboelectric nanogenerator (TENG), nano-friction power generation, wind power, structural models

## Abstract

The escalating global energy demand necessitates the exploration of renewable energy sources, with wind energy emerging as a crucial and widely available resource. With wind energy exhibiting a vast potential of approximately 1010 kw/a per year, about ten times that of global hydroelectric power generation, its efficient conversion and utilization hold the promise of mitigating the pressing energy crisis and replacing the dominant reliance on fossil fuels. In recent years, Triboelectric Nanogenerators (TENGs) have emerged as novel and efficient means of capturing wind energy. This paper provides a comprehensive summary of the fundamental principles governing four basic working modes of TENGs, elucidating the structures and operational mechanisms of various models employed in wind energy harvesting. Furthermore, it highlights the significance of two major TENG configurations, namely, the vertical touch-separation pattern structure and the independent layer pattern for wind energy collection, emphasizing their respective advantages. Furthermore, the study briefly discusses the current strengths of nano-friction power generation in wind energy harvesting while acknowledging the existing challenges pertaining to device design, durability, operation, and maintenance. The review concludes by presenting potential research directions and prospects for triboelectric nanogenerators generation in the realm of wind energy, offering valuable insights for researchers and scholars in the field.

## 1. Introduction

Until recently, fossil fuels such as oil, natural gas, and coal were the primary sources of energy for human production and daily living. The need for fossil fuels has increased in tandem with the fast expansion of human society, and the negative repercussions have progressively surfaced [1,2]. Countries all around the world have banded together in recent years to advocate for low-carbon travel, resource conservation, and the implementation of sustainable development policies. Many governments have begun to actively promote the growth of the electric car sector [3]. As a result, the hunt for alternative energy sources to replace fossil fuels has grown to be critical, and renewable resource research has become a popular issue [4]. Wind energy encompasses the whole globe, including beaches, valleys, deserts, and urban regions. Wind energy, if used wisely, will reduce people’s reliance on fossil fuels.

Triboelectric Nanogenerators (TENG), as an efficient energy harvesting device, has been hotly developed for more than a decade since it was proposed by academician Zhonglin Wang in 2012 [5]. TENG transfers irregular mechanical energy from the environment into electrical energy via the paired action of contact electrification and electrostatic induction [6,7,8]. TENGs are ideal for collecting low-frequency mechanical energy due to their lightweight construction and ease of operation [5]. TENG technology has found widespread use in harvesting renewable energy from natural sources such as wind, ocean, vibration, rain, and human motion [9,10,11,12,13,14].

Given this, researchers have created a succession of Wind-Driven TENGs (WD-TENGs) in the context of wind energy harvesting, continually improving their architectures and updating materials to improve their performance [15,16]. Significant progress has been made in recent years through the use of numerous innovative materials and micro/nanofabrication processes, enhancing TENG-based wind-energy-harvesting technologies. WD-TENG and multifunctional smart sensing systems [17,18] powered entirely by wind-harvested electrical energy have also been created. Extensive research has been committed to achieving higher criteria for wind-energy-harvesting nanogenerators, such as consistent output, appropriateness for difficult conditions [19], extended lifespan, and high power output.

This overview of research gives insights into improvements in friction-based nanogenerators, notably TENG, as efficient wind-energy-harvesting devices. It goes through their operating principles, applications in different renewable energy sources, and the invention of WD-TENGs for wind [20] energy gathering. The incorporation of wind-driven nanogenerators with energy management systems and multifunctional smart sensing systems, as well as the use of innovative materials and micro/nanofabrication processes, are highlighted. The assessment also sheds light on the field’s obstacles and prospects, highlighting the need for wind-energy-harvesting nanogenerators with consistent output, flexibility to diverse conditions, extended lifespan, and high power output.

Wind energy collection using triboelectric nanogenerators is divided into two categories: wind energy collection using a revolving structure and wind energy collection using vibration friction electrification. Wind energy is collected primarily by wind cups, fan blades, and other revolving structures. Wind energy harvesting through vibration and friction electrification involves the generation of electrical energy from wind-induced motion in dielectric materials, predominantly utilizing four types of flow-induced vibration phenomena: vortex-induced vibration [21], galloping [22], flutter [23], and wake gallop [24]. This work systematically describes the most recent developments in wind-energy-based triboelectric nanogenerators, including structural properties, output, and potential future developments. First, the operating principle and method of operation of a TENG are explained, followed by a summary of the materials typically employed in recent years. The technique for developing the structure of a WD-TENG is illustrated from the standpoint of structural optimization, and the typical structure is shown. Furthermore, WD-TENG’s approach to unreliable power production is explored. Finally, present challenges and future development strategies are presented.

## 2. Structural Design and Power Generation Principal Analysis of ST-TENG

### 2.1. Basic Theory of the TENG and the Working Principle

**Figure 1:**
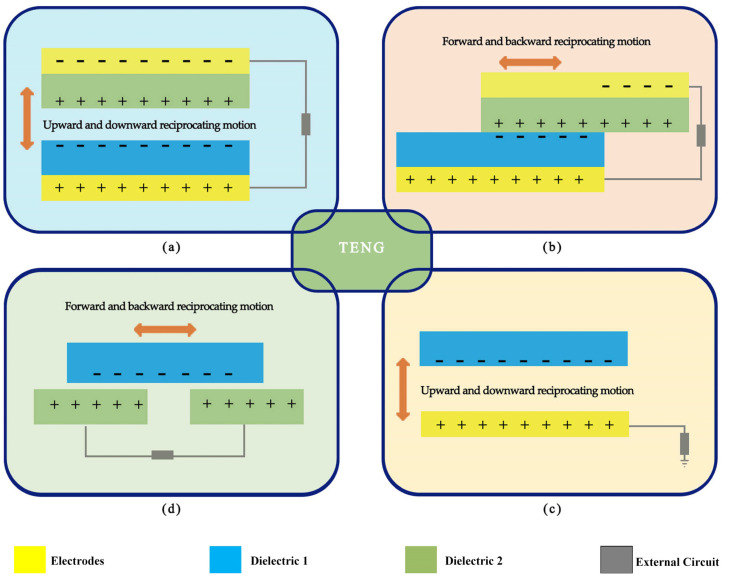
Four basic modes of TENG. (**a**) vertical contact-separation mode, (**b**) lateral sliding mode, (**c**) single-electrode mode, and (**d**) independent triboelectric layer mode.

Triboelectric nanogenerators are self-powered energy conversion devices based on triboelectric and electrostatic inductive-coupling phenomena [25]. There are discrepancies between the two materials’ capacity to receive electrons [26]. When the two materials come into contact, electrons transfer to the material with high electric potential. At the moment, the two are facing separate accusations [27]. When the two elements are adequately spaced, they create both an electrostatic field and a potential difference between them. These two components, connected via an electrode–load–electrode system, respond to regular external driving potential differences. Electrons are driven by this potential difference to flow between the electrodes, seeking to balance the electrostatic potential disparity between the two materials. This continuous contact and separation give rise to alternating current, causing the triboelectric nanogenerator’s output terminal to emit alternating current pulse signals and thereby supply electrical energy externally [28,29,30]. Therefore, the reasonable use of a TENG can convert unharnessed renewable energy into a sustainable source of electricity to meet our power needs. At present, the main working modes of a TENG mainly include the vertical contact separation mode (Figure 1a), lateral sliding mode (Figure 1b), single electrode mode (Figure 1c), and independent triboelectric layer mode (Figure 1d) [31].

### 2.2. The Principle of Static Electricity

**Figure 2:**
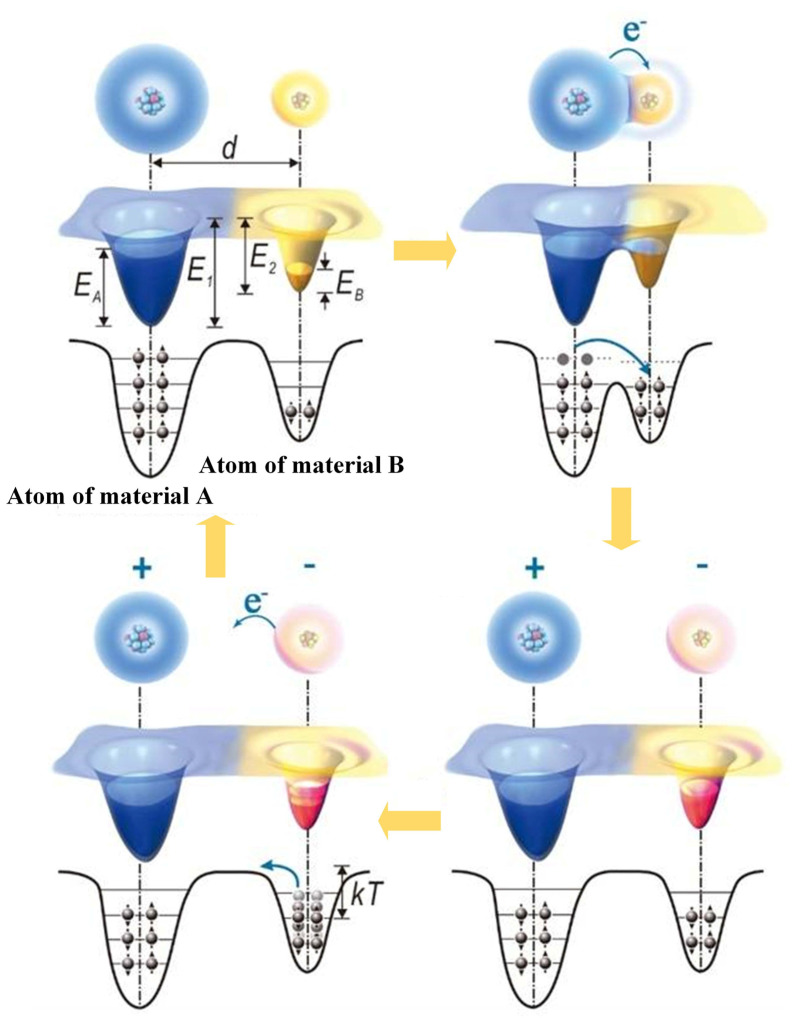
The electronic cloud barrier model of solid–solid contact electrification [35].

The principal process of charge transfer between two contact surfaces is electron transfer [32,33,34]. Wang’s [35,36,37] “electron cloud potential well” model is employed to describe the intuitive contact electrification occurrence between materials (Figure 2). The “electron cloud” is made up of atoms at certain locations in space and electrons in specific electron orbits in molecules. Atoms can be represented by potential wells with loosely bound exterior electrons. The distance between the “electron clouds” is given by *d*. *E*_A_ and *E*_B_ are the electron occupancy energies of materials A and B, respectively, whereas *E*_1_ and *E*_2_ are the energies necessary for electrons from materials A and B to escape. *E*_A_ and *E*_B_ are both smaller than *E*_1_ and *E*_2_. Electrons can only travel about the nucleus before the two materials come into contact, owing to potential well restrictions, and electrons cannot transfer freely. When two different materials come into contact, electron transfer and contact electrification occur only when the distance between their atoms is less than the equilibrium distance, leading to an overlap of electron clouds and the formation of an asymmetric double potential well. This allows atoms in one material (A) to lose electrons, which are captured by atoms in the other material (B). Consequently, B gains electrons, resulting in a negative charge, whereas A becomes positively charged. This phenomenon persists at optimal temperatures, whereupon separation of the materials leads to a majority of transferred electrons remaining with B. However, electron energy fluctuation increases with temperature, leading to the possibility of some electrons returning to A or escaping into the air, thereby weakening the contact electrification event.

### 2.3. Friction Layer Material

**Figure 3:**
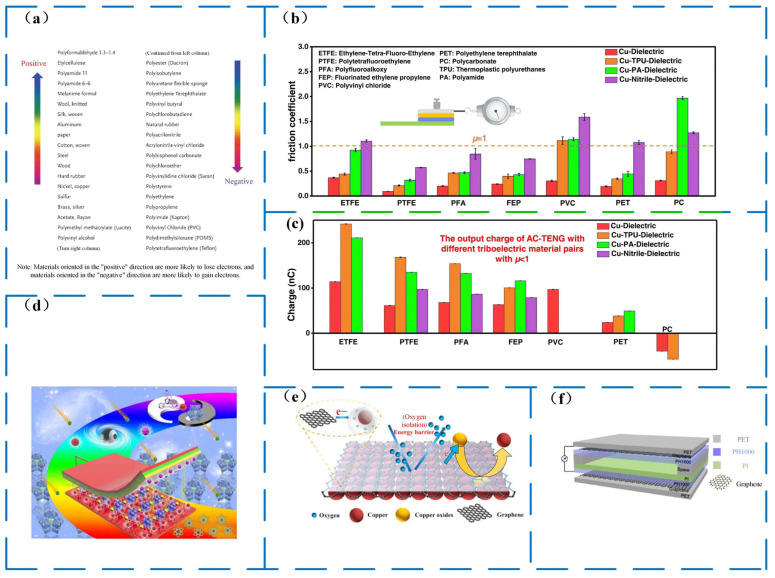
Tribolayer material. (**a**) Triboelectric behavior and triboelectric performance of the TENG under different triboelectric material pairs of common material triboelectric sequence [35]. (**b**) Friction coefficient (μ) between various triboelectric material pairs [43]. (**c**) Effect of different triboelectric materials on the output charge of the TENG [43]. (**d**) Triboelectric mechanism of porous crystalline materials [51]. (**e**) Graphene is impermeable [52]. (**f**) Graphene and graphene composites can be used as TENG auxiliary materials [53].

The material of the friction layer is the key to triboelectrification, so the selection of the material of the friction layer is very critical when designing triboelectric nanogenerators; the effect of contact electrification depends on the polarity of the material, and the polarity of the friction material refers to the material’s ability to capture electrons. The triboelectric sequence of typical materials can be used to guide the selection of friction materials (Figure 3) [35]. Currently, in the field of triboelectric nanogenerators, electrodes are widely composed of dielectric polymers (such as polytetrafluoroethylene (PTFE), polyvinylidene fluoride (PVDF), polydimethylsiloxane (PDMS), nylon, and Kapton) and metallic materials (such as Au, Cu, and Al) [38,39,40,41,42]. The output performance of a TENG depends significantly on the friction coefficient (μ) and the transfer charge. In a study by Cui et al. [43], they used thermoplastic polyurethane (TPU), polyamide (PA), and nitrile as the top friction layer, whereas materials like ethylene-tetrafluoroethylene (ETFE), polytetrafluoroethylene (PTFE) [44,45,46], polyfluoroalkoxy (PFA), fluorinated ethylene propylene (FEP) [47,48,49], polyvinyl chloride (PVC), polyethylene terephthalate (PET) [50], and polycarbonate (PC) were considered representative materials for obtaining electrons in TENG and thus used as the bottom friction layer. Copper was employed as a relative reference material in constructing the metal–dielectric interface. The results of this study can be observed in Figure 3b,c. However, with the development of triboelectric nanogenerators, there are higher requirements for their performance. It is difficult for the main organic polymers to be functionalized and modified to meet more complex applications. Porous crystalline materials (metal–organic frameworks (MOFs), covalent organic frameworks (COFs) with unique tunable structures [51], and carbon derivatives have good electron capture properties as active electrodes. MOFs and COFs are porous crystalline materials. The characteristic of the material enables precise tuning of the electronic structure of the material to optimize the TENG output performance (Figure 3d). Graphene and graphene composite materials are also commonly employed in triboelectric generators as a result of extensive research into carbon components. Graphene has a flexible mechanical behavior and impermeability (Figure 3e) [52], and graphene has a higher oxidation resistance, as well as good electrical and thermal conductivity. Graphene and graphene composite materials can be used as nano-triboelectric generator electrodes, friction materials, and auxiliary materials (Figure 3f). Incorporating natural materials like cotton as the friction layer in a triboelectric nanogenerator designed to harness wind energy results in an increase in both contact area and surface charge density. Using natural cotton as the friction layer material, Xia et al. [53] created a cotton-based triboelectric nanogenerator (C-TENG). The prepared C-TENG can obtain an output performance of 782 V, 8.9 μA, and a peak power of 1.89 mW under optimal conditions.

## 3. Wind Energy Model Based on Triboelectric Nanogenerators

Through mathematical modeling and simulation, triboelectric nanogenerators are convenient in wind energy, and the research cycle and experiment cost are shortened.

For the rotation-based triboelectric nanogenerators, the aerodynamic model of the wind turbine under no-load conditions is expressed as [54]:

(1) $$J\ddot{\alpha }+C\dot{\alpha }+fr=\frac{P_{in}}{\dot{\alpha }}$$

*P_in_* is the mechanical efficiency of the motion input in air is the moment of inertia, α is the rotation angle, *C* is the equivalent damping of the system, *f* is the friction force between the friction layer materials, and *r* is the rotation radius of the contact surface when the two friction layer materials are in contact [55].

(2) $$P_{in}=\frac{1}{2}\rho A\nu^{3}\chi$$

where *ρ* is the density of air (*ρ* = 1.29 kg/m^3^), *A* is the area swept by the rotor blades, *ν* is the velocity of the wind blades, and *χ* is the power coefficient of the rotor.

(3) f=μF

where *μ* is the coefficient of friction, and *F* is the normal force of the friction material’s contact surface.

The transferred charge can be approximated as a sinusoidal function [56], as shown in (4):

(4) $$R_{T}\frac{dQ_{SC}}{dt}=\frac{\sigma \;sin\;8\alpha }{c}-\frac{Q_{SC}}{c}$$

where *Q_SC_* and *R_T_* transfer charge and load resistance, respectively. *c* is the capacitance of two adjacent electrode films, and *σ* is the surface charge density.

The short-circuit current and transferred charge of the vibration-based nanotribogenerator can be derived from the equivalent capacitance model of the TENG as follows [57]:

(5) $$V_{OC}=\frac{\sigma x\left(t\right)}{\varepsilon_{0}}$$

(6) $$Q_{SC}\frac{S\sigma x\left(t\right)}{d_{0}+x\left(t\right)}$$

(7) $$I_{SC}=\frac{dQ_{SC}}{dt}=\frac{S\sigma v\left(t\right)}{{\left(d_{0}+x\left(t\right)\right)}^{2}}$$

where *V_OC_* is the open-circuit voltage, *x*(*t*) is the time-dependent distance between the two friction layers, *I_SC_* the short-circuit current, *Q_SC_* is the transferred charge, *S* is the size of the effective contact area, *v*(*t*) is the change speed of the vibrating plate, *σ* is the triboelectric surface charge density, and *d*_0_ is the thickness of the dielectric layer, defined as *d*_0_ = Σ*d_i_*/*ε_i_*, *ε*_0_, representing the dielectric constant of a vacuum.

## 4. Application of Triboelectric Nanogenerators in Wind Energy Harvesting

Wind energy is created everywhere around us [58,59], but it is not captured and is often lost. TENG can gather wind energy extremely smartly and become a promising technology to harness the energy of the ignored breeze in our lives. Triboelectric nanogenerators for wind energy harvesting primarily comprise wind energy harvesting based on spinning structures and wind energy harvesting based on vibration friction electrification [60]. Following that, standard and unique triboelectric nanogenerators topologies from recent years will be introduced.

### 4.1. Wind Energy Harvesting Based on Rotating Structure

#### 4.1.1. Wind Energy Harvesting Based on Rotating Structure for AC Discharge

**Figure 4:**
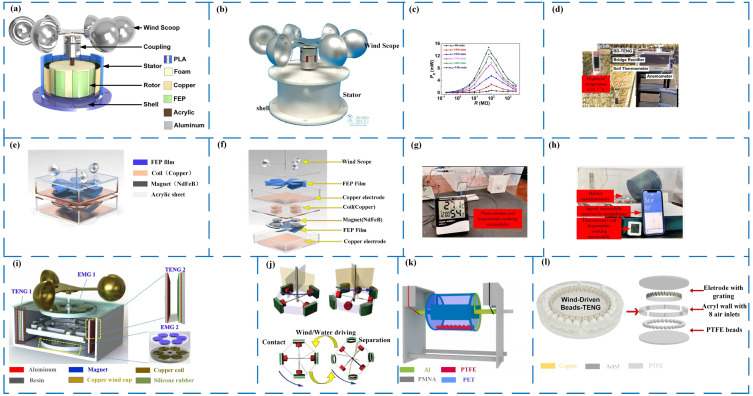
Wind energy harvesting based on rotating structure for AC discharge. (**a**) Schematic diagram of BD-TENG structure. (**b**) The BD-TENG physical map [61]. (**c**) Peak power of BD-TENG at different input speeds [61]. (**d**) The BD-TENG can harvest natural breeze energy to power a soil thermometer [61]. (**e**) Diagram of the 3D model composed of a TENG and an EMG [62]. (**f**) The exploded view of the 3D model is composed of a TENG and an EMG [62]. (**g**) The TENG and EMG are composed of nanotriboelectric generators to power temperature and humidity sensors [62]. (**h**) The TENG and EMG compose a hybrid generator building real-time sensing systems [62]. (**i**) Schematic diagram of TEHG [63]. (**j**) Demonstration and schematic illustration of a magnetically assisted non-contact TENG [64]. (**k**) Schematic illustration of RTS-TENG [65]. (**l**) Schematic (left) and layout sketch (right) of the fabricated WB-TENG [66].

Wind energy harvesting based on a rotating structure performs AC discharge, which is the most basic type of triboelectric nanogenerator used for wind energy harvesting, and other triboelectric nanogenerators used for wind energy harvesting are generated on this basis. Its structure is quite simple as compared to other wind power generators based on rotation, and its power generation efficiency is relatively objective. Its structure is getting increasingly innovative as researchers continue to investigate it in depth. The sealed triboelectric nanogenerators that capture wind energy are more practical in usage and more adaptable to the environment in which it is utilized. To achieve high conversion efficiency, low-density materials, particularly fan blades and rotors, should be used as much as feasible. In 2022, Li et al. [61] developed the BD-TENG, a breeze-driven triboelectric nanogenerator (as illustrated in Figure 4a,b). It has a sealed construction and low-density materials. When the wind speed is 4 m/s, the energy conversion efficiency of BD-TENG can reach 12.06%; the output performance is 330 V, 7 μA, and 137 nC; and the peak power is 2.81 mW. Still quite impressive (Figure 4c), BD-TENG successfully powered the soil thermometer (Figure 4d). The frictional nanogenerator is not a separate subject, it can be combined with other forms of power generation to improve power generation efficiency. Ying and his group designed a hybrid frictional nanogenerator in 2022 (as shown in Figure 4e,f), a hybrid nanogenerator composed of a TENG and an EMG; at a wind speed of 6 m/s, the peak power of the TENG could reach 0.38 mW, and the peak power of the EMG could reach 118 mW, which could be used to drive small electronic devices and micro-sensing systems (such as Figure 4g,h) [62]. Fan et al. designed a wind-driven triboelectric-electromagnetic hybrid nanogenerator, TENG (Figure 4i) [63]. The maximum output powers of the TENG and EMG were 0.36 mW and 18.6 mW, respectively.

Magnetic-assisted technology is also well suited to TENG. Mechanical movement and magnetism are used to separate contacts. As early as 2016, Huang Longbiao et al. developed a magnetically assisted non-contact triboelectric nanogenerator for wind energy and blue energy, the collector (Figure 4j) [64] with a *V_OC_* peak of about 206 V and an *I_SC_* of 30 µA at 6 Hz.

Triboelectric nanogenerators can be designed with two different structures for collecting wind energy: one where the inner ring acts as the rotor to rotate, and another where the outer ring serves as the rotor while the inner ring remains fixed or consists of free particles. This construction is mainly used to gather ocean energy, although it may also be used to harness wind energy. The more intricate internal structure is removed by this structure. The construction is basic, but it demands a lot of driving force. In 2022, Zhu’s team created a triboelectric nanogenerator RTS-TENG (Figure 4k) with an outer ring revolving, and its *V_OC_* and *I_SC_* could reach 144 V and 1.23 µA, respectively [65]. Jin Dayuan et al. designed a triboelectric nanogenerator with the rolling motion of magnetic beads as a wind energy harvester WB-TENG as early as 2018 (Figure 4l) [66]. When the wind speed was 10 m/s, an open circuit voltage of 18.5 V could be generated, and the short-circuit current could reach 2.3 μA. When the wind speed was 20 m/s, the peak power density of WB-TENG was 1.36 mW/cm^2^.

Wind energy harvesting primarily relies on a rotating structure for AC discharge. In this process, wind energy is transformed into mechanical energy [67], which drives the rotation of dielectric materials or electrodes. This rotation disrupts the initial electrostatic balance, leading to the generation of electrical energy, which results in relatively high output power, as shown in Table 1. EMG-TENG hybrid structure combines the advantages of the two and has higher output power compared to other forms of triboelectric nanogenerators [68], but the addition of magnets increases the overall mass of the device, which limits its flexibility. The overall mass of the device limits its flexibility, the pure TENGs require less driving energy, the triboelectric nanogenerators with orb particles have no advantage in terms of output compared to the traditional wind-cup type of triboelectric nanogenerators (e.g., Figure 3a vs. Figure 3b), and the removal of the wind-cup from the structure simplifies the structure.

#### 4.1.2. Wind Energy Harvesting Based on Rotating Structure for Direct Current Discharge

If DC power is desired, ordinary nano-friction power generation must include a rectifier circuit to convert AC power to DC electricity. The triboelectric nanogenerators based on wind energy collection based on rotation, on the other hand, may transform wind energy into DC power via a basic construction, boost discharge power, and power tiny equipment. A novel approach to save space in the power supply system.

**Figure 5:**
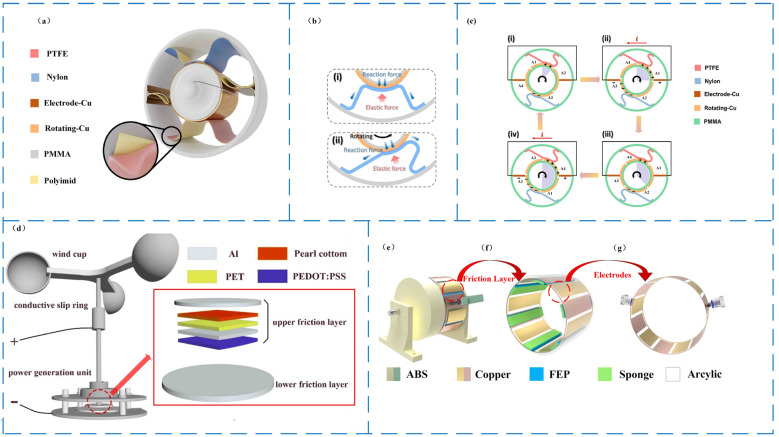
Wind energy harvesting based on rotating structure for DC discharge. (**a**) Schematic diagram of wind-driven DAS-TENG nanogenerator [69]. (**b**) Schematic diagram of the total force exerted on the arched film [70]. (**b**) (**i**) shows the total force applied to the rotating cylinder and arch-shaped triboelectric film. (**ii**) As the inner substrate rotates, the film is deformed by pushing in the direction of rotation. (**c**) The working mechanism and output of the schematic diagram [68]. (**d**) Schematic diagram of the RTS-TENG [70]. (**c**) (**i**) When the inner substrate rotates 45 degrees. (**ii**) When the inner substrate rotates 90 degrees. (**iii**) When the inner substrate rotates 135 degrees. (**iv**) When the inner substrate rotates 180 degrees. (**e**) Structural design diagram of BD-TENG [74]. (**f**) Friction generator set [74]. (**g**) A partially enlarged photo of the mechanical rectifier [74].

Song Minghuan et al. designed a deformable, arched-film-based DC triboelectric nanogenerator (DAS-TENG) in 2023 for current amplification to collect wind energy [69] (Figure 5a). The total force applied on the revolving cylinder and the arching triboelectric film is shown in Figure 5b(i). The thin film is distorted by being pressed in the direction of rotation when the inner substrate is rotated, as illustrated in Figure 5b(ii). This design’s power generation is quite clever. Figure 5c depicts the procedure of producing direct current. The entire procedure is a complete closed loop. The ingenious layout causes the charge to travel in the same direction continually, and the current output generates a direct current in the same direction. The DAS-TENG produces a peak V overclock output of 300 V and a peak closed-circuit current output of 1.7 A. The DAS-TENG structure of this design is very novel. It utilizes the elasticity of the friction material to increase the contact area and improve the output efficiency. It can output DC without rectifying current and has a simple structure. To enhance power generation performance and achieve direct current (DC) output, researchers developed an innovative organic semiconductor/metal Schottky DC triboelectric nanogenerator (DC-TENG). By employing organic semiconductors and metals as the friction layer, this new design significantly reduces impedance, leading to improved power supply efficiency compared to conventional triboelectric nanogenerators. In 2023, Zhong Yuanyou and colleagues introduced the windmill RTS-TENG [70], which embodies the Schottky heterojunction concept. The structure of the windmill is depicted in Figure 5e, with a friction layer consisting of PEDOT: PSS and Al. Through Schottky contact and the tribovoltaic effect, the windmill generates direct current in the external circuit, achieving *V_OC_* (open-circuit voltage) of 0.6 V and *I_SC_* (short-circuit current) of 3.6 μA when exposed to a wind speed of 7 m/s.

The aforementioned triboelectric nanogenerators generate direct current by virtue of their structure and material qualities. Brushes can also be used in the field of TENG: [71,72,73]. A report by Guang and colleagues in 2022 shows a bidirectional DC triboelectric nanogenerator, BD-TENG, with a mechanical rectifier (Figure 5e) [74]. The BD-TENG is made up of mechanical structural components, a triboelectric generating unit (Figure 5f), and a mechanical rectifier (Figure 5g). The BD-TENG can generate an open-circuit voltage of 480 V and a short-circuit current of 12 A, and it can also power a calculator reliably at a wind speed of approximately 7 m/s.

Currently, various methods exist for converting alternating current into direct current, with the bridge rectifier being the most commonly employed approach to achieve stable DC output [75]. This method has found widespread use in self-powered sensing systems; however, its application in the field of a TENG is limited due to the power consumption associated with the bridge rectifier, which adversely affects TENG’s output performance (refer to Table 2). To improve TENG’s output performance, a promising approach involves optimizing the structure to separate electrons and subsequently induce their flow through the external circuit for neutralization. Additionally, leveraging the unique properties of semiconductor materials and incorporating brushes in TENG designs have been shown to enhance output performance as well.

#### 4.1.3. Construction of Self-Regulating Triboelectric Nanogenerators for Wind Energy Harvesting

The self-adjusting structure can adjust the distance between friction materials and control friction based on the external wind speed, resulting in more stable and high-power electric energy that is conducive to driving under breeze; this self-adjusting mechanism has high power generation performance, its frictional resistance and material wear are reduced, and its equipment performance and service life are significantly improved.

**Figure 6:**
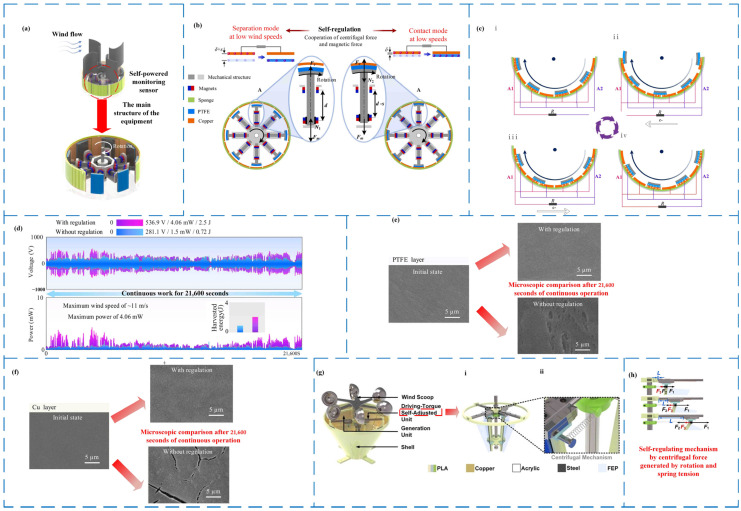
Self-regulating triboelectric nanogenerators for wind energy harvesting. (**a**) Schematic diagram of the TENG-SS structure [56]. (**b**) The working mechanism of a self-regulatory system [56]. (**c**) The working principle of TENG-SS (from i to iv represents one cycle) [56]. (**i**) Assuming the sector plate is coincident with electrode aluminum in the initial state. (**ii**) As the sector plate slides from electrodes A1 to A2. (**iii**) The sector plate is coincident with electrode A2. (**iv**) As the sector plate slides from electrodes A2 to A1. (**d**) The voltage and power of the prototype with a self-regulating strategy and the prototype without a self-regulating mechanism driven by natural wind for 21,600 s [56]. SEM images of (**e**) PTFE film and (**f**) copper film after continuous operation for 21,600 s with and without an adjustment mechanism [56]. (**g**) Schematic diagram of the structure of SA-TENG (**i**) driving torque self-adjusting unit, (**ii**) four centrifugal mechanisms) [76]. (**h**) Forces acting during operation, centrifugal force F_1_, spring tension F_2_, static friction F_3_, and stretched length L of the spring [76].

In 2022, Zou’s team. developed a self-regulating triboelectric nanogenerators TENG-SS for a self-powered wind speed sensor (Figure 6a) [56]. TENG-SS is made up of blades, rotating shafts, telescoping arms, and electrode rings. Two magnetic bodies that repel each other are the key to its adjusting process. Figure 6b depicts the adjustment process (where δ denotes the degree of contact, and s represents the separation distance). When the wind speed exceeds the critical value, they will make contact; when the wind speed falls below the critical value, they will separate. In this case, the wearing of parts is decreased to some extent. Figure 6c depicts the charge transfer between A_1_ and A_2_ during the sliding motion of its fan-shaped plate. It features eight generating units, which means it creates power eight times faster than typical single-producing triboelectric nanogenerators. Under the same conditions, the output performance of the TENG equipped with a self-regulating mechanism is higher than that of the TENG without a regulating mechanism, as shown in Figure 6d, and the self-regulating mechanism significantly reduces the wear of the friction layer material (Figure 6e), improves its service life, and allows it to be used in more complex environments. TENG-SS has a max voltage of 558 V and an average power of 2.79 mW. In addition to the magnetic force approach, simpler parts, such as springs, can be employed. Consequently, Wang et al. created a driving torque self-regulating triboelectric nanogenerator SA-TENG in 2022 that efficiently collects random wind energy (Figure 6g) [76]. The four centrifugal mechanisms (Figure 6g(ii)) in the driving torque self-regulating unit (Figure 6g(i)) are in charge of regulating it. At varying wind speeds, the force acting on the SA-TENG during operation (Figure 6h) may be modified by adjusting the interaction between the centrifugal force F_1_, the spring tension F_2_, the static friction force F_3_, and the spring stretching length (L). In another instance, Wang et al. conducted a comparison between ordinary triboelectric nanogenerators (N-TENG) without a self-regulating structure and electromagnetic generators (EMG) under identical conditions. The results revealed that the charging capacity of the self-regulating triboelectric nanogenerator (SA-TENG) was 2.3 times higher than that of the N-TENG and an impressive 18.7 times greater than the EMG.

As shown in Table 3, the TENG-SS, which uses the magnetic properties of the magnet, is more advantageous in terms of the required drive energy, whereas the SA-TENG has a good output performance, and, overall, there are advantages in self-regulating triboelectric nanogenerators over other rotating triboelectric nanogenerators in terms of output performance and the service lives of nano-friction generators. The self-regulating triboelectric nanogenerators with self-regulating mechanisms extend the service life significantly. The core principle of the self-adjusting triboelectric nanogenerators is to incorporate force-outputting components, such as elastic materials like springs and rubber bands, as well as magnets. These additions serve a dual purpose: during light wind conditions, they reduce the distance between the two materials of the friction layer, consequently minimizing friction force and lowering the required driving energy, leading to improved output performance. When the wind speed is strong, the friction layer is touched for charging, and the contact area is raised to receive the greatest output performance optimization. In broad terms, the self-adjusting triboelectric nanogenerators reduce friction layer wear and extend service life.

### 4.2. Wind Energy Harvesting Based on Vibration Triboelectricity

Wind energy harvesting using vibrating plate friction electrification is primarily based on the contact separation method. Triboelectric nanogenerators generate electricity through the vibration of the dielectric material between the electrodes, which offers energy-saving advantages compared to the rotating mode [77]. Additionally, these generators are characterized by their simplicity in size, manufacturing process, and high driving efficiency. At low wind speeds, this system proves to be highly efficient in collecting wind energy. However, it has a limitation in that it can only harness wind energy effectively from one direction in general.

**Figure 7:**
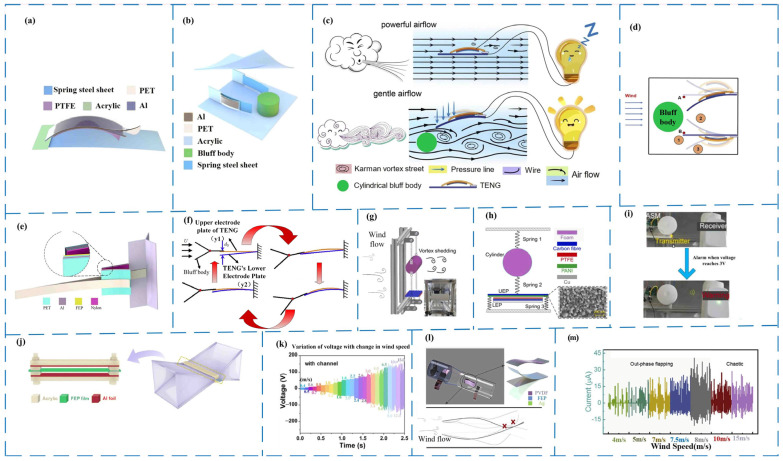
Harvesting wind energy with a vibration-based flake nano tribogenerator. (**a**) Structure of WG-TENG generator [78]. (**b**) The 3D schematic diagram of the wind-energy-harvesting device composed of WG-TENG [78]. (**c**) Schematic diagram of the flow field with and without cliff bodies [78]. (**d**) (1), (2), and (3) schematically represent the detailed motion state of the cantilever beam structure in the flow, whereas points A and B are free cantilever tips located symmetrically on both sides of the cliff body [78]. (**e**) Schematic diagram of GTENG [80]. (**f**) Simplified diagram of GTENG work [80]. (**g**) The 3D schematic of VIV-TENG [81]. (**h**) The 2D structural model of VIV-TENG [50]. (**i**) VIV-TENG powers the wireless alarm sensor [81]. (**j**) Schematic of the W-TENG structure after adding channels [82]. (**f**) Output voltage at different wind speeds after adding channels [83]. (**l**) B-TENG harvesting wind energy system [83]. (**m**) Output current of triboelectric nanogenerators with different flow rates [83].

For the utilization of wind energy to create vibration power production in the power generating unit, an auxiliary is often included to establish a wake phenomenon, making it simpler to drive and enhancing efficiency. Yuan Lei et al. [78] created a triboelectric nanogenerator, WG-TENG, based on the phenomena of wake galloping to capture wind energy in 2022. WG-TENG is a simple friction nano-generator that operates in the contact-separation mode. As shown in Figure 7b, a cylinder is employed as the cliff body, and two WG-TENG power-generating units are fitted symmetrically with the cliff body to increase the power generation performance. When the sheet TENG is put in a consistent wind flow, the vibration is minimal, and the frequency instability phenomena of airflow are more common when the wind speed is high [79]. However, in the case of low wind speed, or even light wind, by introducing a bluff body into the mechanism in the flow field, a wake galloping phenomenon (as shown in Figure 7c) is generated, causing the device to oscillate. We can understand that the wind is due to the appearance of obstacles entering and then entering a relatively spacious space; the wind is suddenly released, complex movement changes occur, and the pressure difference between the inlet and outlet causes the mechanism to vibrate, showing excellent potential in breeze energy harvesting. Figure 7d depicts the detailed motion state of WG-TENG. The WG-TENG, created by Yuan and colleagues, has a high sensitivity in terms of wind speed inspection structure and can operate at wind speeds as low as 1 m/s. It has a simple construction, good mobility, cheap cost, and high conversion efficiency, and it offers significant promise for large-scale breeze energy harvesting. Regarding the use of the galloping phenomenon, Zhang et al. designed a galloping triboelectric nanogenerator GTENG (as shown in Figure 7e) [80] for energy harvesting at low wind speeds as early as 2020, using an independent cantilever beam structure and mechanical knowledge. The V mechanism is used to collect wind energy to generate up and down vibration (Figure 7g). When the wind speed is 4 m/s, a peak voltage of 400 V is produced.

The vortex-induced vibration of fluid is also a common method of inducing vibration. In addition, adding an elastic structure can increase the vibration frequency of the vibration-based triboelectric nanogenerators to a certain extent. In 2022, Zhang and their team embarked on a quest using vortex-induced vibrations to design a vortex-induced vibration triboelectric nanogenerator, VIV-TENG, for low-speed, wind-energy-harvesting (as shown in Figure 7g,h), which had high average power [81]. When the wind speed was at 2.78 m/s, it could generate an average power of 392.72 μW and an average power density of 96.79 mW/m^2^, which could readily power wireless alarm sensors (Figure 7i) and wireless monitoring sensors, which is useful for powering wireless sensor networks (WSN) installed in remote places.

Along with the methods mentioned above for increasing wind energy collection efficiency, it is also possible to increase wind energy collection by expanding the channel, increasing wind speed and density through the method of collecting large openings and inputting small openings and increasing the vibration frequency to increase power generation. Quite recently, Zhu’s team harnessed low-speed breeze wind energy and a wind-driven triboelectric nanogenerator (W-TENG) mounted inside a square variable-diameter channel (construction illustrated in Figure 7j) [82]. The largest improvement was observed in the output performance without additional channels, which was 11 times higher. It was capable of absorbing wind energy at speeds ranging from 0.4 m/s (output voltage of 2 V) to 15 m/s (as illustrated in Figure 7k). The Bernoulli effect could also be used to induce the vibration of the dielectric material. The Bernoulli effect is a statement of the relationship between the flow velocity and the pressure in the fluid system. Emerging endlessly, the way of collecting wind energy has been expanded. In 2022 [83], Chen Xin et al. prepared a triboelectric nanogenerator, B-TENG, that used the Bernoulli effect to collect wind energy (as shown in Figure 7l). Remarkably, as the wind speed increased, the B-TENG exhibited a proportional enhancement in its output performance, reaching its maximum potential at 8 m/s, with impressive values of 175 V voltage, 43 μA current, and 2.5 mW power (Figure 7m).

The three-dimensional triboelectric nanogenerators for wind energy harvesting utilize a sheet power generation unit connected to another three-dimensional moving component, thereby altering the structure of the sheet-head power generation unit to achieve a three-dimensional configuration. This design offers a larger contact area with the wind, resulting in improved power production performance. However, it should be noted that three-dimensional triboelectric nanogenerators have a relatively larger volume, involve more intricate construction, and incur fewer costs compared to conventional two-dimensional systems.

**Figure 8:**
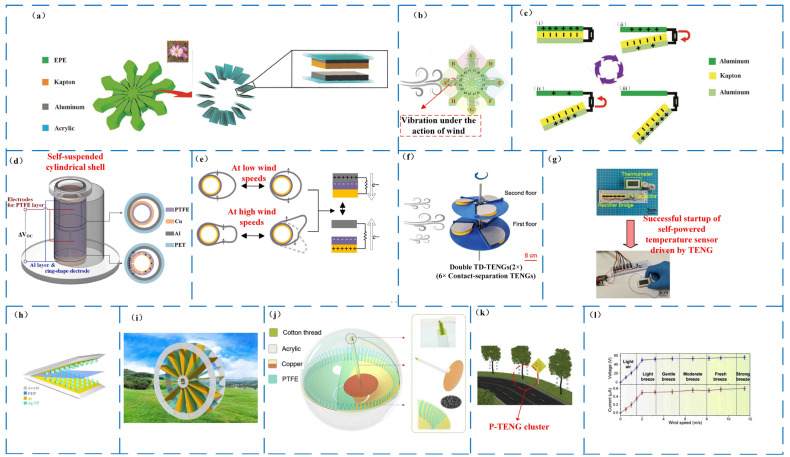
Vibration-based three-dimensional nanotribogenerator harvesting wind energy. (**a**) Schematic diagram of the triboelectric nanogenerator structure of Phalaenopsis [84]. (**b**) Schematic diagram of the Phalaenopsis-structured triboelectric nanogenerator measuring wind direction and magnitude [84]. (**c**) Phalaenopsis-structured triboelectric nanogenerator generating electricity [84]. (**i**–**iv**) TENG under the action of wind force in the wind from the initial contact state to the gradual separation until the maximum separation state is reached, and then gradually re-contact. (**d**) Self-suspended shell-based triboelectric nanogenerator for wind energy harvesting [85]. (**e**) Power generation principle of a self-suspending shell-based triboelectric nanogenerator [85]. (**f**) Schematic diagram of a bilayer TD-TENG based on six TENG units [86]. (**g**) Photograph of the TD-TENG self-powered temperature sensing system [86]. (**h**) The device structure of the AS-TENG [87]. (**i**) Schematic of the integrated AS-TENG array [87]. (**j**) Schematic illustration of the P-TENG [88]. (**k**) Schematic illustration of a P-TENG cluster serving as a traffic light to power LEDs [88]. (**l**) The relationship between the output voltage of the P-TENG and the wind speed [88].

Triboelectric nanogenerators can also be involved in the field of bionics, where inspiration can be obtained, and novel structures can be designed. A design on a triboelectric nanogenerator C-TENG based on the Phalaenopsis structure was made based on the state of the petals in the wind by Zhao as displayed in Figure 8a [84]. It can be used to measure the wind direction and size (Figure 8b). Taking the EPE petal as shown in Figure 8b as an example, A can swing left and right under the action of wind energy to form contact and separation with the fixed electrode, and the power generation process is based on a1; for example, Figure 8c. In the design, Zhao shows that multiple power generation units are formed, which is also relatively accurate for identifying the direction of the wind. When the wind speed is 15 m/s, the C-TENG device generates an *I_SC_* = 0.34 μA, *V_OC_* = 61.7 V, and the peak power density is 11.7 mW/m^2^, however, it is worth noting that the device’s jitter frequency under the influence of wind remains relatively low.

Similarly, Gao proposed a self-suspended shell-based triboelectric nanogenerator [85] for omnidirectional wind energy harvesting as presented in Figure 8d for a better method of creating significant jitters. The cylinder improves the contact area for gathering wind. Figure 8c depicts the power-generating principle. It can capture wind energy at speeds ranging from 0.3 to 10 m/s. When the low wind speed approaches 3 m/s, the output voltage steadily continues to grow, and, when the wind speed reaches 10 m/s, the RMS voltage saturates at 47.68 V.

The above-mentioned structure can be simplified as a vertical separation mode placed perpendicular to the horizontal plane. This structure occupies a larger space than the horizontally placed structure. Gao’s design of a turbine disk for wind energy collection and self-powered wildfire warning served as a triboelectric nanogenerator TD-TENG (as shown in Figure 8f) [86], and its structure is equivalent to the vertical separation mode placed parallel to the horizontal plane. The design is suitable for the operating conditions of low wind speed near the ground. When the load is 7 MΩ, the output performance is 230 V open circuit voltage, 9 μA short-circuit current, the transferred charge is 82 nC, and the maximum peak power is 0.37 Mw. It can be used as a low-power electronic device. The distributed power supply is shown in Figure 8g. Quite recently, Lin and other researchers developed an angular triboelectric nanogenerator, AS-TENG (Figure 8h) [87], for collecting environmental wind energy. After the integrated array (Figure 8i), the integrated device can deliver an open-circuit voltage (*V_OC_*) of about 120 V and a short-circuit current (*I_SC_*) of about 40 μA under the wind speed of 25 m/s. The simple pendulum effect generated by the use of vortex-induced vibration is also one of the inspirations for TENG to generate electrical energy. Inspired by the single pendulum phenomenon in 2019, Lin et al. also designed an ultra-sturdy and frequency-doubling triboelectric nanogenerator, P-TENG (Figure 8j) [88]. Multiple P-TENGs can be connected in series to form a power generation network as street lights and other equipment are powered (as shown in Figure 8k). When the wind speed of P-TENG is greater than 2 m/s, the maximum output voltage of P-TENG is about 56 V (Figure 8l).

In summary, based on the vibration friction initiation of wind energy collection, based on the vibration friction initiation of wind energy collection of TENG, mainly contact separation type, can be utilized by the instability of the wind energy itself, including the speed of the wind, the force generated by the action on a certain surface, and so on. It is also possible to artificially expand its instability by adding some special structures to increase the vibration effect. The most prominent feature of assembling multiple power generation units into a three-dimensional device or increasing the contact area of individual power generation units through changing their shapes, for example, rolling them into cylinders, is that it realizes the full use of space and improves the output power of a TENG. As shown in Table 4, the most prominent feature of TENGs for wind energy collection based on vibration friction initiation is that they require less start-up energy, can collect breeze energy, and, in general, the output performance of the three-dimensional type is better than that of the lamellar type among TENGs for vibration-based wind energy collection. The output energy of the rotary type is higher than that of the vibratory type among TENGs for wind energy collection, but the power density of the rotary type is lower than that of the vibratory type. Diverse natural structures in nature are one of the sources of inspiration for designers, and, in bionic design, we mimic the structural movements of TENGs to the creatures in nature in order to realize structural innovations. Although TENG has many advantages, its disadvantages are also very evident: small power generation, power generation stability, etc. Through structural innovation, we find the optimal structure, so the bionic TENG has a huge development space in the future.

## 5. The Factors That Can Modify Triboelectric Nanogenerators to Improve Performance

Triboelectric nanogenerators take up less area, are more environmentally friendly [89], have lower production costs [90], and are more adaptable [91]. Despite continuous improvements in their output performance, they do have certain limitations, such as unpredictable power generation.

### 5.1. Output Stability

**Figure 9:**
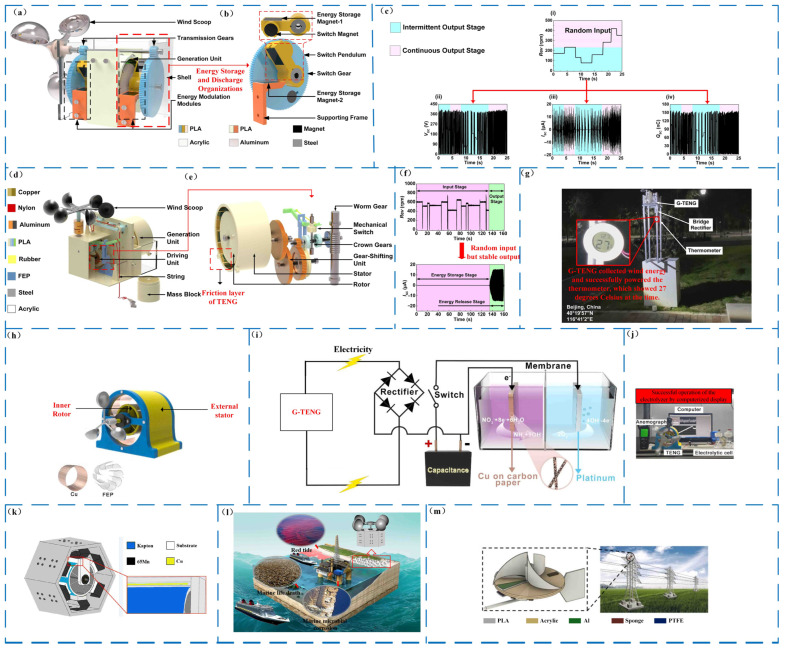
The solution to the problem of unstable power generation. (**a**) Schematic diagram of the overall structure of a magnetic switch-structured triboelectric nanogenerator (MS-TENG) [95]. (**b**) The MS-TENG energy modulation module [95]. (**c**) Output performance of the MS-TENG under random wind speed [95]. (**i**) Random input. (**ii**) Open circuit voltage. (**iii**) Short-circuit current. (**iv**) Transfer charge. (**d**) Schematic illustration of the overall structure of the G-TENG [96]. (**e**) Assembly relationship among mechanical components. (**f**) The output performance of G-TENG under random rotation [96]. (**g**) The G-TENG power supply for small appliances [96]. (**h**) The basic structure of the RL-TENG [97]. (**i**) Schematic diagram of the wind-driven electrochemical NRA system [97]. (**j**) A picture of the self-powered electrocatalytic NRA system [97]. (**k**) Schematic diagram of the R-DC-TENG mode l [98]. (**l**) Possible applications of R-DC-TENG self-powered electrolysis [98]. (**m**) Schematic diagram of the structure and application scenarios of the WM-TENG [99].

It is quite evident that most of the output performance of the frictional nanogenerator is related to the input of external wind energy. The higher the wind speed, the greater the output power, and the input voltage of ordinary sensors is generally constant at about 5 V. However, ensuring its output voltage and power stay the same, especially at low wind speeds, is a challenge. For this problem, an external transformer can be used to ensure voltage stability, but the transformer can only ensure that high voltage changes to low voltage and cannot realize low voltage to high voltage, so as to improve the ability of wind energy collection; the energy required for driving should be reduced as much as possible, and the collection range should be increased. Additionally, an external energy storage device, such as a battery, is necessary to store the triboelectric nanogenerators to collect excess electric energy generated by high wind speed [92,93,94]. Under low wind speed, the energy storage device discharges or stores wind energy into other forms of energy and, subsequently, releases it stably to generate electrical energy. Liu’s design of a triboelectric nanogenerator with a magnetic switch structure for continuous and periodic harvesting of wind energy MS-TENG demonstrates this in Figure 9a [95]. It uses magnetic bodies to store and release energy (Figure 9b). Two bilateral magnetic bodies pass through gears under the action of wind energy, and the distance between the magnetic bodies is continuously reduced. When the distance reaches a critical value, the connected magnetic bodies are separated. The scaled energy is transferred through a one-way bearing; Liu verified by experiments that when the input speed is higher than the critical speed, its output is continuous and regular (as depicted in Figure 9c), with an open circuit voltage of 410 V, a short-circuit current of 18 μA, and a 155 nC. The output performance of the transferred charge and the peak power were 4.82 mW. Wang’s team also designed a gravitational triboelectric nanogenerator G-TENG in 2021, as seen in Figure 9d,e [96], which was used to stably collect natural wind energy. It uses mechanical transmission to convert wind energy into mechanical energy. When the crucial value is achieved, the mechanical switch is actuated, the heavy item is released, and the power-producing unit is powered up to create electricity. TENG’s steady output and performance are evaluated using standard deviation (*I_SO_*) and fluctuation degree (IFD). Even with random wind speed input, the triboelectric nanogenerator exhibits a relatively stable current output (Figure 9f). The peak value of the short-circuit current, characterized by the standard deviation (*I_SO_*), remains below 0.31 μA, resulting in a fluctuation degree (IFD) of 2.3%. Notably, this stable performance enables the successful powering of small electrical appliances, as demonstrated in Figure 9g.

The electrical energy generated by the triboelectric nanogenerators is directly used for the refined substances. Wang et al. designed a lightweight triboelectric nanogenerator-driven, self-powered electrocatalytic nitrate ammonia production method in 2023 [97]. They designed a roller-shaped TENG (RL-TENG) (Figure 9h); RL-TENG was combined with the NRA system, a power management circuit composed of rectifiers and capacitors was connected in the circuit (Figure 9i,j), and the traditional NH, compared with other methods, had considerable potential and evident advantages, such as convenience, sustainability, and environmental protection. The wind energy harnessed by triboelectric nanogenerators is also mostly used to gather ocean wind. The energy generated by gathering sea wind can be directly used for marine pollution treatment. The use of civil voltage directly affects objects and human activities. Generally, it needs to be processed before it can be used. In the process of transmission, the cost of voltage processing is extremely high, the ability to harness wind energy generated by the frictional nanogenerator can be used directly, and the power is also relatively suitable. Another design by Liu in 2022 [98] featured a wind-driven rotating direct current. Triboelectric nanogenerators (Figure 9k) are used for the self-powered inactivation of seawater microorganisms (Figure 9l). When the wind speed is 10 m/s, the peak value of the circuit voltage can reach 450 V, the peak value of the short-circuit current can reach 11 μA, and the matching impedance is 1 MΩ; R-DC-TENG can generate a direct current, and electrolysis can produce chlorine and hypochlorite. To achieve the effect of inactivating microorganisms in the sea area around the coast and islands, one should produce 8.60 mg/L of available chlorine within minutes. The triboelectric nanogenerators can also act as a sensor for wind speed and wind direction, instead of as a power source for power generation, and use the electrical signals generated by it at different wind speeds and wind directions to achieve a certain measurement effect. In 2022, Tang Xiaolong et al. introduced a novel application of nano-friction power generation to harness wind energy for sensing purposes. They devised a self-powered wind sensor called WM-TENG (as depicted in Figure 9m), based on triboelectric nanogenerators [99]. This WM-TENG sensor was specifically designed to detect the vibrations of the breeze on transmission lines. Tang Xiaolong and the research team successfully demonstrated that WM-TENG could effectively serve as a wind-vibration-monitoring tool for transmission lines when integrated with a specially designed detection system.

### 5.2. Output Performance of a TENG for Harvesting Wind Energy

Presently, the overall output performance of a WD-TENG is relatively small, limiting its ability to only drive low-power electronic devices. The most critical parameter for WD-TENG is its output performance. To enhance this aspect, increasing the contact area proves to be effective, although it necessitates consideration of wear issues. Additionally, optimizing the structure, reducing friction in the friction layer material, elevating the frequency of movement [100,101,102,103], and refining the output performance are crucial steps. Building a TENG power generation network can bring about a qualitative change, achieved through optimizing the connection structure with rigid connections. This synchronous motion of the power generation TENG group significantly improves its output performance. Moreover, cable optimization is essential, as connecting multiple WD-TENGs with cables may lead to reduced output performance due to increased resistance. Optimizing the structure, the addition of elastic elements and magnetic elements to increase the movement frequency inside the TENG (wool, fabrics, etc.) to increase the electrostatic effect inside the TENG can also absorb the collisions generated by the internal parts of the TENG during the movement and reduce internal consumption. Using the collision structure should be avoided when designing the structure, so as to improve the output performance

### 5.3. Lifetime of a TENG Applied to Harvest Wind Energy

In order to improve the performance of friction nanogenerators, attention should not be limited to their output performance but also to their lifetime, which is an important performance parameter. The key to the application of the TENG that collects wind energy is its lifespan. The service life is one of the decisive factors for its application in practice and is a key element that needs to be improved. The practical application environment of frictional nanogenerators that collect wind energy in the future is expected to be complex and harsh. For these nanogenerators to thrive in such conditions, their maintenance, inspection, and replacement must be carefully considered to ensure an extended service life. Addressing these challenges necessitates a holistic approach that encompasses durability, charge transfer capability, and output performance consolidation. Implementing self-regulating mechanisms can reduce friction and mitigate material wear, further enhancing the nanogenerator’s longevity. Additionally, optimizing the structure, employing rolling friction between materials in the internal friction layer, and utilizing high-performance materials are crucial steps toward providing reliable and efficient wind energy collection in challenging environments. By focusing on these aspects, frictional nanogenerators can realize their full potential and meet the demands of future applications. Use materials with high fatigue strengths, explore the availabilities of these high-fatigue-strength materials in friction nanogenerators, and enrich the selection of materials. The molding process of materials should also be continuously optimized to control the molding temperatures of materials, speeds, and other processing parameters; optimize the mechanical properties of the materials through micro-nano processing technologies; reduce the roughness of the instant noodles; reduce the friction coefficients; and increase service lives [104,105,106]. The shell of the TENG that collects wind energy is made of materials with sufficient and high enough strength, and the overall structure adopts a modular and combined structure, which is convenient for later maintenance and replacement.

## 6. Conclusions and Prospect

Wind energy is a type of sustainable energy that is widely distributed and has vast reserves. Reasonable harvesting will aid in changing the current energy structure, particularly in our period dominated by electricity, and our contemporary vital vehicles such as automobiles. It is also increasingly moving toward electric driving, and the triboelectric nanogenerators have a lot of promise for gathering breeze. The development of TENG, which is based on the connection of contact charging with electrostatic induction, has opened up new possibilities for microenergy technology and self-power supply systems. This study focuses mostly on the innovative structure obtained for a period of time, with the objective of assisting future academics in structural innovation. This paper also discusses the power-generating concept and fundamental mode of nano-triboelectric generators, as well as certain specific materials. More materials will be added to the triboelectric nanogenerator in the future as a result of researchers’ ongoing exploration, and several inadequacies in the triboelectric nanogenerator are noted, as well as alternatives.

Triboelectric nanogenerators offer a plethora of advantages, including high output power, excellent conversion efficiency, versatility in material selection, ease of fabrication, cost-effectiveness, and lightweight design. As a result, they hold immense potential for application in various domains, notably energy harvesting and information sensing. However, oriented to practical applications, triboelectric nanogenerators still have a very large space for development, and there are still many shortcomings to be further studied and explored in the future. It is mainly reflected in the output power, the stability of output, and the service life under adverse conditions. For power generation and service life, the fundamental factor lies in the characteristics of the friction layer and electrode materials, but TENG electrodes and friction layer material optimizations are less explored, and people should optimize the material properties, create new materials, and boldly try new structures and new materials to improve the triboelectric nanogenerators’ power generation and conversion efficiencies and service lives. At present, the development of a friction nanogenerator relies on the uncertainty of the deep mechanism, mainly through the experimental analysis of the performance of a TENG. In order to improve the efficiency of the design of a TENG, the physicochemical mechanism of the TENG’s design optimization strategy, and explore the basic theory of modeling, the parameter of the quantization of the research needs to be further explored.

TENG has enormous application potential in wind energy harvesting in the future as a revolutionary clean energy technology. It is predicted to become a key method of harvesting clean energy and will make a significant contribution to solving energy concerns. Future research priorities include fully examining wind-energy-harvesting factors, developing a generic model theory of wind energy harvesting, and simplifying its mathematical formulae. Millions of TENG units will be connected by cables to form a power generation group. This power generation group can collect wind energy near the coast, above the mountains, at the tuyere of the valley, and in daily life. The wind energy triboelectric nanogenerators can also be combined with other generators, such as solar, EMG, etc., to obtain the maximum possible energy and improve the robustness of continuous energy harvesting. The TENG Power Generation Group can store the generated electric energy in a battery energy management system to supply power for small- and medium-sized equipment in daily life and engineering. Millions of TENG units will be linked by wires to form a power-producing group. This power-generating group may gather wind energy along the ocean, above the mountains, at the valley tuyere, and in everyday life. The wind energy triboelectric nanogenerators may also be coupled with other generators, such as solar, EMG, and so on, to acquire the most energy and increase the robustness of continuous energy harvesting. The TENG Power Generation Group may store the generated electric energy in a battery energy management system to power small- and medium-sized equipment in everyday living and engineering.

## Figures and Tables

**Table 1:** TENG performance of wind energy harvesting based on rotating structures for AC discharge in recent years.

Author	TENG	Starting Wind Speed	Maximum Wind Speed	Open-Circuit Voltage (V)	Short-Circuit Current	Power Density and Power
Li et al. [61]	BD-TENG	3.3 m/s	12 m/s	330	7 µA	2.81 mW
Ying et al. [62]	Triboelectric-electromagnetic Nanogenerator	2.6 m/s	6 m/s	84	40 mA	118.38 mW
Fan et al. [63]	TEHG	4 m/s	15 m/s	479.2	1.9 mA	18.96 mW
Huang et al. [64]	Magnetic-assisted Noncontact Triboelectric Nanogenerator	2 Hz	6 Hz	206	30 µA	3 mW
Zhu et al. [65]	RTS-TENG		5 m/s	144	1.23 µA	
Jin et al. [66]	WB-TENG	3 m/s	10 m/s	18.5	2.3 µA	

**Table 2:** TENG performance of rotating-structure-based wind energy harvesting for DC discharge in recent years.

Author	TENG	Starting Wind Speed	Maximum Wind Speed	Open-Circuit Voltage (V)	Short-Circuit Current	Power Density and Power
Song et al. [69]	DAS-TENG	4 m/s	6 m/s	300	1.7 A	633.44 mW
Zhong et al. [70]	RTS-TENG	2 m/s	7 m/s	0.6	3.6 μA	0.78 mW/m^2^
Guang et al. [74]	BD-TENG		7 m/s	480	12 μA	0.96 W/m^2^

**Table 3:** Output performance of the TENG designed with self-regulating function for wind energy harvesting in recent years.

Author	TENG	Starting Wind Speed	Maximum Wind Speed	Open-Circuit Voltage (V)	Short-Circuit Current	Power Density and Power
Zou et al. [56]	TENG-SS	2 m/s	12 m/s	558 V		2.79 mW
Wang et al. [76]	SA-TENG	5 m/s	13.2 m/s	500 V	21 μA	7.69 mW

**Table 4:** Output performance of TENG for vibration-based harvesting of wind energy over several years.

Author	TENG	Starting Wind Speed	Maximum Wind Speed	Open-Circuit Voltage (V)	Short-Circuit Current	Power Density and Power
Yuan et al. [78]	WG-TENG	1 m/s	8.1 m/s	350 V	6 μA	149 mW/m^2^
Zhang et al. [80]	GTENG	1 m/s	2 m/s	220 V	7 μA	4.3 μW
Zhang et al. [81]	VIV-TENG	1.66 m/s	2.78 m/s	450 V	29 µA	4.8 mW
Zhu et al. [82]	W-TENG structure after adding channels	0.4 m/s	15 m/s	150 V		0.6 mW
Chen et al. [83]	B-TENG	1.6 m/s	8 m/s	175 V	43 μA	2.5 mW
Zhao et al. [84]	C-TENG		15 m/s	61.7 V	0.34 μA	11.7 mW/m^2^
Gao et al. [85]	Self-suspended shell-based triboelectric nanogenerator	0.3 m/s	10 m/s	47.6 V		8.43 mW/m^2^
Gao et al. [86]	TD-TENG			230 V	9 µA	0.37 mW
Lin et al. [87]	Individual AS-TENG	4 m/s	25 m/s	120 V	40 µA	0.82 mW
Lin et al. [88]	Individual P-TENG	0 m/s	2 m/s	56 V	0.5 μA	

## Data Availability

The data that support the findings of this study are available from the corresponding author, upon reasonable request.

## Abbreviations

**Abbreviation**	**Expansion**
TENG	Triboelectric nanogenerators
BD-TENG	Breeze-driven triboelectric nanogenerator
TEHG	Triboelectric-electromagnetic hybrid nanogenerator
RTS-TENG	Real-time signal output terminal structure Triboelectric nanogenerators
WB-TENG	Triboelectric nanogenerator based on rolling motion of beads
DAS-TENG	Deformable, arch-shaped, film-based direct-current triboelectric nanogenerator
RTS-TENG	Organic semiconductor/metal Schottky heterojunction based direct current triboelectric nanogenerator
BD-TENG	Bidirectional direct current triboelectric nanogenerator with the mechanical rectifier
TENG-SS	Self-regulation strategy for triboelectric nanogenerator
SA-TENG	Driving-torque self-adjusted triboelectric nanogenerator
WG-TENG	Wake-galloping-driven triboelectric nanogenerator
GTENG	Gaslloping triboelectric nanogenerator
VIV-TENG	Vortex-induced vibration triboelectric nanogenerator
W-TENG	Wind-powered triboelectric nanogenerator
B-TENG	Bernoulli effect-dominated triboelectric nanogenerator
C-TENG	Calliopsis-inspired biomimetic triboelectric nanogenerator
TD-TENG	Unique turbine disk-type triboelectric nanogenerator
AS-TENG	Angle-shaped triboelectric nanogenerator
P-TENG	Pendulum triboelectric nanogenerator
MS-TENG	Magnetic-switch-structured triboelectric nanogenerator
G-TENG	Gravity triboelectric nanogenerator
RL-TENG	Roller-shaped TENG
R-DC-TENG	Direct-current triboelectric nanogenerator
WM-TENG	Wind-monitoring triboelectric nanogenerator
